# C1 Compound Biosensors: Design, Functional Study, and Applications

**DOI:** 10.3390/ijms20092253

**Published:** 2019-05-07

**Authors:** Jin-Young Lee, Bong Hyun Sung, So-Hyung Oh, Kil Koang Kwon, Hyewon Lee, Haseong Kim, Dae-Hee Lee, Soo-Jin Yeom, Seung-Goo Lee

**Affiliations:** 1 Synthetic Biology & Bioengineering Research Center, KRIBB, Daejeon 34141, Korea; jylee84@kribb.re.kr (J.-Y.L.); bhsung@kribb.re.kr (B.H.S.); sohyungoh@kribb.re.kr (S.-H.O.); kkkwon@kribb.re.kr (K.K.K.); hlee@kribb.re.kr (H.L.); haseong@kribb.re.kr (H.K.); dhlee@kribb.re.kr (D.-H.L.); 2 Department of Biosystems & Bioengineering, KRIBB School of Biotechnology, University of Science & Technology, Daejeon 34113, Korea

**Keywords:** C1 compound biosensor, genetic enzyme screening system, one-carbon compound

## Abstract

The microbial assimilation of one-carbon (C1) gases is a topic of interest, given that products developed using this pathway have the potential to act as promising substrates for the synthesis of valuable chemicals via enzymatic oxidation or C–C bonding. Despite extensive studies on C1 gas assimilation pathways, their key enzymes have yet to be subjected to high-throughput evolution studies on account of the lack of an efficient analytical tool for C1 metabolites. To address this challenging issue, we attempted to establish a fine-tuned single-cell–level biosensor system constituting a combination of transcription factors (TFs) and several C1-converting enzymes that convert target compounds to the ligand of a TF. This enzymatic conversion broadens the detection range of ligands by the genetic biosensor systems. In this study, we presented new genetic enzyme screening systems (GESSs) to detect formate, formaldehyde, and methanol from specific enzyme activities and pathways, named FA-GESS, Frm-GESS, and MeOH-GESS, respectively. All the biosensors displayed linear responses to their respective C1 molecules, namely, formate (1.0–250 mM), formaldehyde (1.0–50 μM), and methanol (5–400 mM), and they did so with high specificity. Consequently, the helper enzymes, including formaldehyde dehydrogenase and methanol dehydrogenase, were successfully combined to constitute new versatile combinations of the C1-biosensors.

## 1. Introduction

One-carbon (C1) compounds, such as methane, methanol, CO, CO_2_, and formate are promising starting chemicals with the potential to become valuable renewable resources [1,2,3,4,5]. Many research groups have focused on the biological conversions of C1 gases using native microorganisms, such as methanotrophs, *Clostridium,* and *Acetobacterium* for CH_4_, CO_2_, and CO, respectively [6,7].

Another critical challenge is the development of new recombinant enzymes for C1 gas assimilation [8]. Many of the heterologous enzymes or redesigned pathways have been tested in *Escherichia coli*, to assess the use of methanol as a carbon source [9,10,11,12]. There was also an attempt to use formate and CO_2_ in the presence of glycine [13]. Despite these efforts, no recombinant *E. coli* using C1 compounds as the sole carbon source have yet been identified, thereby demonstrating the need for effective tools to develop C1-converting enzymes.

**Figure 1:**
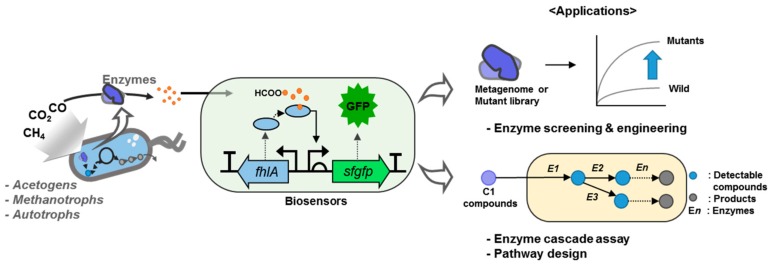
The importance of biosensors for enzyme screening or new pathway development in a C1 gas biorefinery. In the schematic figure of biosensor, the solid arrows and dotted arrows represent promoter and the translation of the respective genes, respectively.

Given the recent advances in synthetic biology, the transcriptional regulator (transcription factor [TF])-based biosensor has emerged as one of the most powerful tools to increase enzyme activity or to discover new enzymes or strains. Various TF-based biosensors have been developed for enzyme engineering and the discovery of novel biocatalysts, using a fluorescence-activated cell sorting (FACS)-based high-throughput screening system [14,15,16,17,18,19,20,21]. Formaldehyde- and formate-dependent TFs have been reported in the operon of formaldehyde detoxification and the expression system of anaerobic formate dehydrogenase, respectively [22,23]. However, to the best of our knowledge, a specific TF for methanol, which might be a representative C1 compound, has not yet been reported. The absence of C1 compounds related to TFs can be overcome by incorporating helper enzymes that can transform an undetectable molecule into a molecule that can be detected by an existing or developed biosensor from an enzymatic reaction [17,24]. Using this biosensor, it is possible to search not only for new enzymes, but also for new pathways in the C1 gas bioconversion field. More specifically, it is possible to discriminate the enzymatic activity related to C1 metabolism, new enzyme screening, design pathways, or to study the enzymatic cascade reactions by measuring the yield of a product using a combination of an enzyme reaction and a biosensor (Figure 1).

In this study, we developed TF-based biosensors to detect formate (FA-GESS), formaldehyde (Frm-GESS), and methanol (MeOH-GESS), which are major chemicals in C1 gas assimilation pathways. Using these biosensors, we detected C1 chemicals (formate, formaldehyde, and methanol) and the activities of enzymes, such as formaldehyde dehydrogenase and methanol dehydrogenase, which are involved in C1 metabolism. Moreover, the biosensors incorporating the C1-converting enzymes could offer “user-selectable” starting substrates through the designed enzymatic reactions. These biosensors are powerful tools for the screening of C1-converting enzymes and new pathways in C1 gas assimilation.

## 2. Results and Discussion

### 2.1. Design and Construction of the C1 Biosensors

The biosensors used in this study are listed in Table 1. To develop biosensors that responded to formate, formaldehyde, and methanol, we selected wild-type formate and formaldehyde-responsive TFs and then integrated them into the genetic circuits.

**Figure 2:**
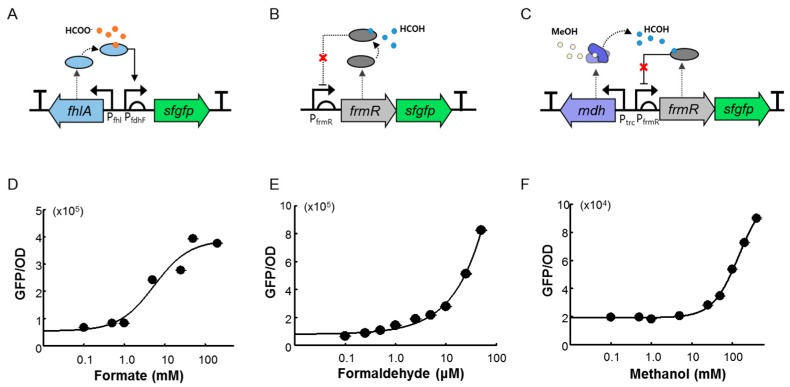
Design and fluorescence responses of the C1 biosensors. (**A**) Formate biosensor was designed using a formate-dependent activator (FhlA) and cognate promoter (PfdhF) with a sfGFP reporter. (**B**) Formaldehyde biosensor was designed using a formaldehyde-responsive regulator (FrmR) and cognate promoter (PfrmR) with a sfGFP reporter. (**C**) Methanol biosensor was designed by incorporating methanol dehydrogenase (Mdh) into the formaldehyde biosensor. For the overexpression of *mdh*, the *trc* promoter was used. The solid arrows and dotted arrows represent promoter and the translation of the respective genes, respectively. The release of repression by the ligand binding in the repressor type of regulatory protein, frmR, represent red X. (**D**) Fluorescence intensities of formate biosensor. Normalized fluorescence was compared at different formate concentrations. (**E**) Fluorescence intensities of formaldehyde biosensor. Normalized fluorescence was compared at different formaldehyde concentrations. (**F**) Fluorescence intensities of methanol biosensor. Normalized fluorescence was compared at different methanol concentrations. All values represent mean ± SD of three independent experiments.

In *E. coli*, the formate dehydrogenase enzyme is encoded by the *fdnGHI* operon, which is positively regulated by *fhlA* as a σ54-dependent transcriptional activator in the presence of formate [22]. Therefore, we examined the applicability of the *E. coli fhlA* regulatory subunit for the development of a formate-detectable biosensor (FA-GESS). The *fhlA* gene was inserted under the control of a native promoter (P_fhl_) in the direction opposite to that of the PfdhF promoter–*sfGFP* fusion sequence (Figure 2A). To develop the formaldehyde biosensor (Frm-GESS), frmR was used as a positive TF of the formaldehyde detoxification operon in the *E. coli* genome. It is a member of the RcnR/CsoR family of metal-sensing transcriptional repressors and responds to formaldehyde [25]. In Frm-GESS, the *frmR* gene was under the control of its native P_frmR_ promoter and *sfgfp* next to the *frmR* gene, like an operon (Figure 2B).

A two-component system based on methanol-sensing *E. coli* showed the DNA-binding activity of MxaX by phosphorylation in response to the presence of extracellular methanol/formaldehyde. However, this sensor cell has no methanol dose dependency because it is a target-nonspecific two-component system [26]. Thus, we incorporated *mdh* from *Bacillus methanolicus* as a helper enzyme into the formaldehyde biosensor to construct MeOH-GESS as an “expanded” target-specific biosensor. Consequently, the detection of methanol was achieved with Mdh, which is known to oxidize methanol to formaldehyde. The *mdh* gene under the control of the *trc* promoter was integrated into Frm-GESS (Figure 2C). Finally, we obtained three versions of the biosensors, namely, FA-GESS, Frm-GESS, and MeOH-GESS.

### 2.2. Quantitative Responses of FA-GESS, Frm-GESS, and MeOH-GESS

To evaluate the characteristics of FA-GESS, Frm-GESS, and MeOH-GESS, the biosensors were expressed in the *E. coli* DH5α cells, and the fluorescence was measured in the presence of different concentrations of formate, formaldehyde, and methanol, respectively. The growth retardations of the sensor cells were observed at concentrations of 250 mM formate, 50 μM formaldehyde, and 400 mM methanol. Given that formaldehyde is particularly cytotoxic to *E. coli*, Frm-GESS cells only grow with 50 μM formaldehyde. For all the biosensors, the increased fluorescence intensity positively corresponded to substrate concentration (Figure 2D–F). In the Frm-GESS, there was no signal in response to the methanol, which implied its applicability to a methanol sensor by incorporating Mdh.

For the formate biosensor, a small increase in the perturbation of the fluorescence signal was observed. Presumably, this might have been caused by the σ-54–dependent transcriptional mechanism, and further study would involve optimization in terms of the genetic circuit, enzyme activity, and reaction conditions. Consequently, the dynamic ranges and minimal detection ranges were calculated (Table 2). The fold-changes in the fluorescence intensities toward formate, formaldehyde, and methanol were 7-, 12-, and 5-fold, respectively, compared to the baseline signal (without substrates). In a previous study, a formaldehyde-responsive biosensor in the C1 gas-related research field was developed with a similar detection threshold [27,28].

**Figure 3:**
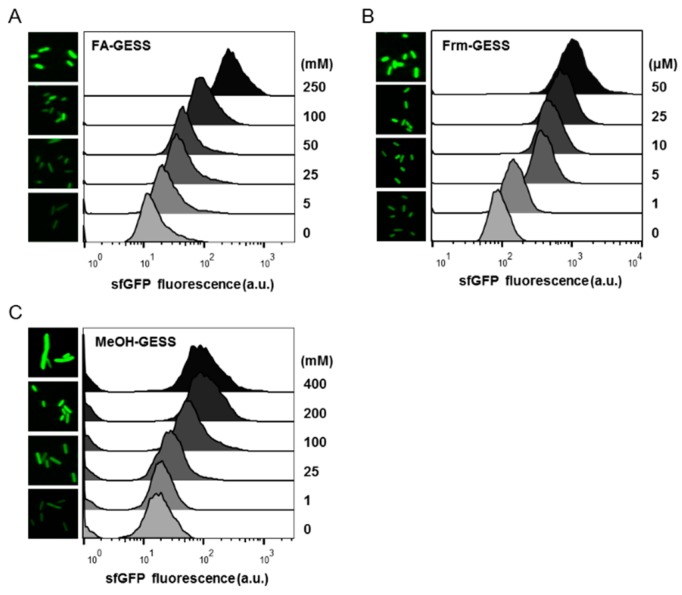
Flow cytometric analysis and microscopy images of the C1 biosensors. (**A**) Formate biosensor. (**B**) Formaldehyde biosensor. (**C**) Methanol biosensor. Fluorescence-activated cell sorting (FACS) and confocal microscopy data show that each of the biosensors functioned well at the single-cell level. In the case of the methanol biosensor, the images show a slight suppression of cell growth at high doses of methanol. For the formate and formaldehyde biosensors, growth retardation was diminished at high doses of substrates.

Cells containing each biosensor were analyzed by flow cytometry in the presence of different concentrations of substrates (Figure 3A–C). Except at the highest concentration of methanol (200 mM), no distinctive differences in terms of the physical change of the cells were observed (Figure 3A–C). Dose-dependent fluorescence signals to each substrate indicated that the biosensors effectively detected all the substrates at the single-cell level. Therefore, the C1 biosensors could be applied to other studies aimed at enhancing enzymatic activity, finding new enzymes in the C1 assimilation pathways, or screening strains by the FACS.

**Figure 4:**
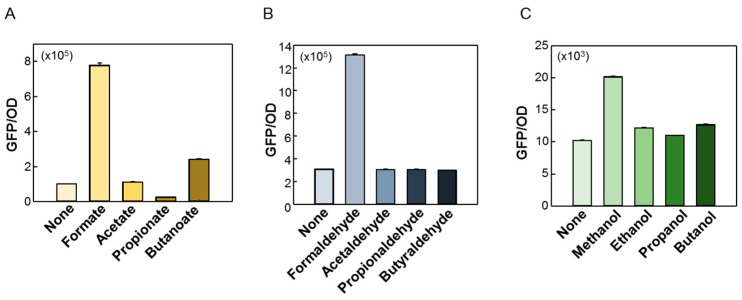
Substrate specificity of the C1 biosensors. (**A**) C1–C4 acids (**B**), aldehydes, and (**C**) alcohols were administered to the respective biosensor cells, and the fluorescence response signals were compared.

The ligand specificity of the C1 biosensors was also examined using C1–C4 carbon acids, aldehydes, and alcohols (Figure 4A–C). Biosensors may interfere with other molecules in the organism owing to the broad substrate specificity of the transcriptional factor. For the formate biosensor (Figure 4A), the formate induced a strong fluorescence response amongst the four substrates, and the butanoate slightly increased the fluorescence signal. For the formaldehyde (Figure 4B) and methanol biosensors (Figure 4C), only the formaldehyde and methanol, respectively, specifically induced the fluorescence responses. These results suggested that each biosensor was compatible because of these substrate specificities, and therefore it was advantageous to find new enzymes in the pathway that did not interfere with each other.

### 2.3. Use of the C1 Biosensor to Detect C1-converting Enzyme Activities

To test the applicability of the C1 biosensors, FalDH and Mdh were used. These enzymes participate in methane metabolism in methanotrophs [29]. Briefly, methane monooxygenases oxidize the methane to methanol, which is then further oxidized to formaldehyde by the Mdh activity. Formaldehyde is a branchpoint metabolite; it can be further oxidized to formate for energy conservation by FalDH or it can be incorporated into the serine or ribulose monophosphate (RuMP) pathway to increase the biomass [29]. These enzymes have attracted attention in the C1 gas assimilation pathway, and are considered good candidates for model studies.

**Figure 5:**
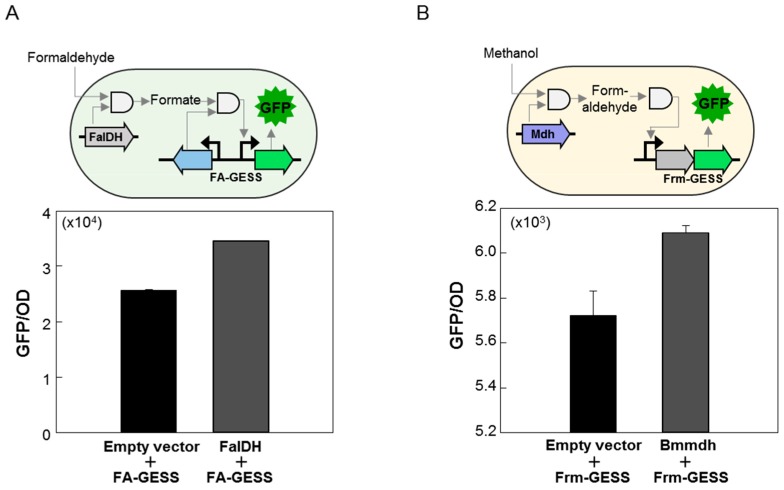
Application study of C1 biosensors. (**A**) Graphical representation of the application of the C1 biosensors with sequential enzymes. (**B**) Activities of the formaldehyde dehydrogenase (FalDH) and methanol dehydrogenase (Bmmdh) were measured using the respective biosensor cells. Three hours after induction with the IPTG (1.0 mM), formaldehyde (100 μM) or methanol (200 mM) were added, and then the fluorescence signal was measured. The solid black arrows represent promoter site. The gray arrows indicate the operational path of biosensor when the substrates and enzymes was added.

*E. coli* expressing pFA-GESS and FalDH from *Pseudomonas putida* or pFrm-GESS and Bsmdh from *B. stearothermophilus,* were used to investigate whether the biosensors could test the activities of the C1-converting enzymes [30,31,32]. FalDH is an NAD^+^-dependent oxidoreductase that reversibly catalyzes formaldehyde to formate. For FA-GESS/FalDH co-expression, the fluorescence signal intensity after the addition of formaldehyde (100 μM) was dependent on the expression of FalDH, suggesting that the FA-GESS biosensor system could effectively measure the FalDH activity (Figure 5A). For Frm-GESS/Bsmdh, the fluorescence intensity increased after the addition of methanol (200 mM) (Figure 5B). These results suggested that the biosensors could effectively monitor the enzyme activity or could detect the starting substrate through an enzymatic reaction. These results also suggested a way to immediately expand the range of biologically detectable C1 chemicals by introducing certain enzymes that transform non-detectable molecules into molecules for which sensors already exist. This is quite important in the field of C1 gas bioconversion where enzyme activity detection and C1 chemical detection are difficult. The biosensor could be used to explore new C1 assimilation pathways in the future.

### 2.4. Perspective of C1-Detectable Biosensors

A living organism has a variety of sensing systems, such as riboswitches, TFs, enzymes, and promoters that tightly regulate gene expression in response to changes in the environmental stimuli. Research on the development of biosensors using small molecules has been widely conducted using these native sensing systems, and consequently various biosensors have been developed [33].

For the development or engineering of useful enzymes, the utilization of the TF-based biosensors is regarded as a very effective strategy. Previously, we also developed biosensors for cellobiose, phenol, lactam, and terpenoid, for use in enzyme engineering or the screening of libraries to identify novel enzymes, where we successfully developed enzymes that have new functions or enhanced activity through the FACS device-based high-throughput screening system [14,15,16,17,18,19]. However, only a formaldehyde-responsive biosensor in the C1 gas-related research field has been developed [27,28].

Although the information regarding the TFs that respond to C1 compounds is insufficient, the biosensors developed in this study showed adequate sensor activity to discriminate the enzyme activity of Mdh and FalDH (Figure 5A,B). However, it was still necessary to enhance the sensor activity owing to the high background and low sensitivity. In recent studies, formaldehyde biosensors were improved through the modification of the promoter sequence and its DNA secondary structure [27,28]. This result implied that the performance of the biosensors could be improved by changing parts of the biosensor. Therefore, further studies will need to optimize the performance of biosensors via a combination of bio-parts and DNA secondary structures, as well as the engineering of TFs.

As mentioned previously, the advantages of biosensors are that they can analyze activity in real-time and at the single-cell level. Therefore, a large library of samples could be effectively explored using high-speed analytical instruments such as FACS. Another advantage is that the biosensors could be used in combination, thereby possibly allowing for the simultaneous exploration of various compounds. Recently, a 12-compound-sensing biosensor cell called Marionette *E. coli* was developed by reducing the background signals and cross-talk, and by increasing the dynamic range and sensitivity [34]. This biosensor cell could be used as a tool to analyze the enzyme cascades or for pathway development. Additionally, these biosensor systems can be applied in *P. putida* with various advantages for natural product biosynthesis, such as a versatile intrinsic metabolism with diverse enzymatic capacities and an outstanding tolerance to xenobiotics [35]. For these reasons, the genetic circuit-based sensor in *P. putida* should be further studied.

**Figure 6:**
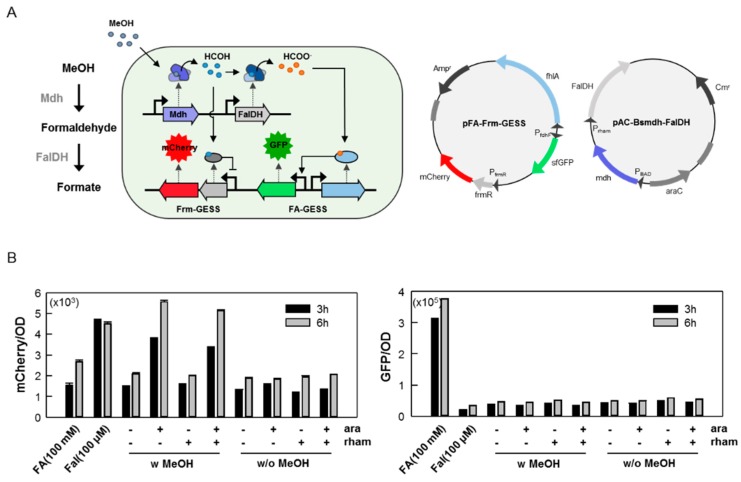
Application of the C1 biosensors to study the enzyme cascade in recombinant cells. (**A**). Schematic view of the operation of the combined C1 biosensor according to the respective enzymatic activity, and the structure of the recombinant vectors used in this study. For recombinant vectors, each sensor modules simply combined by Gibson-cloning method, and the expression of Mdh and FalDH was under controlled by PBAD and Prham promoter, respectively. (**B**). Each biosensor modules was well functioned, independently, when combined each other. The mCherry or sfGFP fluorescence signals was only obtained when the respective substrates were added. Formaldehyde produced by Mdh was detected by Frm-GESS sensor (mCherry signal, left graph), but the formate produced by FalDH was not detected by FA-GESS sensor (sfGFP signal, right graph). All the experiments began with induction (0.2% arabinose, 0.1 mM rhamnose) to express the enzymes during 1 h, followed by the addition of methanol (200 mM). Fluorescence signals were obtained after 3 or 6 h.

In this study, the formate and formaldehyde biosensors were independently operational because of the different transcriptional regulators. Accordingly, these biosensors could be applied simultaneously in a single cell, enabling us to monitor not only the related enzymatic activity but also the enzymatic cascade through simply combining each sensor module (Figure 6A). In this case, the fluorescence signal would be changed depending on the enzymatic activities of the Mdh and FalDH, which could be monitored by adding a specific inducer. As a result, mCherry, which directly correlated with Mdh activity, exhibited fluorescence, but gfp, induced by FaldH activity, did not show a fluorescence signal (Figure 6B). The reason was that this conversion only led to very small amounts of products, less than 1 mM, and thus the detection of formate produced by FalDH from formaldehyde could not be achieved. This could be solved by improving the detection limit or increasing the activity of the FalDH in a future study. We suggest that this strategy is applicable to study the strain development or pathway engineering related to C1 assimilation.

## 3. Methods

### 3.1. Materials

All the reagents used in this study were purchased from Sigma-Aldrich (St. Louis, MO, USA). The restriction endonucleases, polymerases, and DNA cloning kits were obtained from New England BioLabs (Ipswich, MA, USA), and the DNA purification kits were from Promega (Madison, WI, USA). Oligonucleotides and synthesized genes were provided by Macrogen (Seoul, Korea) (Table S1). The DNA preparation and manipulation techniques were performed according to standard molecular biology protocols.

### 3.2. Construction of Formate, Formaldehyde, and Methanol Biosensors

The plasmids used for the biosensor in this study are listed in Table 1. The oligonucleotides used are listed in Table S1. The biosensor plasmids were constructed using the Gibson assembly method following the manufacturer’s instructions. To construct the biosensor, pCL-GESS was used as the plasmid backbone containing a reporter and a terminator without TF, and with a cognate promoter as in Reference [17]. For the formate biosensor (FA-GESS), a formate-dependent transcription activator gene (*fhlA*) and its native promoter were amplified using PCR from the genomic DNA of *E. coli* K12. The superfolder GFP gene (*sfgfp*) was used as a reporter and it was subcloned under the hypoxia-inducible promoter PfdhF, which is regulated by FhlA as described in Reference [22]. The TF and reporter genes were located in opposite directions to avoid transcriptional noise. For the formaldehyde biosensor (Frm-GESS), a formaldehyde-responsive TF (*frmR*) gene and its native promoter were amplified by PCR from the genomic DNA of *E. coli* K12. The *sfgfp* gene was subcloned next to the *frmR* gene, consisting of an operon-like structure. The methanol biosensor (MeOH-GESS) was constructed by incorporating the methanol dehydrogenase gene (*mdh*) into the formaldehyde biosensor, and it was designed to detect methanol by an enzyme-coupled reaction. The *Bacillus stearothermophilus mdh* gene was synthesized and subcloned into the formaldehyde biosensor construct with a *trc* promoter as in Reference [36]. The sensor plasmid pFA-Frm-GESS was constructed via PCR amplification of each sensor module and using the Gibson assembly method. The sfGFP of the Frm-GESS module was replaced by mCherry. The expression vector pAC-Bsmdh-Faldh was constructed using pACBB [37] as a backbone plasmid, and the expression of Bsmdh and FalDH was controlled by P_BAD_ and P_rham_ promoters, respectively.

### 3.3. Fluorescence-Based Functional Analysis of C1 Biosensors

Each recombinant *E. coli* DH5α strain harboring pFA-GESS, pFrm-GESS, and pMeOH-GESS was cultured in LB or M9 minimal medium containing the appropriate antibiotics (ampicillin 50 µg/mL) at 37 °C for 10 h with various concentrations of formate (0.1–250 mM), formaldehyde (0.1–100 μM), and methanol (0.1–400 mM). The other chemicals (treated with 200 mM acids and alcohols, and 50 μM of aldehydes) were also tested to confirm the ligand specificity.

To confirm the ligand-activated GFP signal, fluorescence intensity was measured using a multi-label microplate reader (PerkinElmer, Waltham, MA, USA) at excitation and emission wavelengths of 485 and 535 nm, respectively. The fluorescence intensity of each of construct at the single-cell level was measured using a FACS Calibur system (BD Biosciences, San Jose, CA, USA). In the FACS, a blue laser (488 nm) and band-pass filter (530/30 nm) photo multiplier tube detector were used to analyze the fluorescence signals at the single-cell level. Flow cytometric data were analyzed using the FlowJo software package (FlowJo, Ashland, OR, USA). The Hill equation was used to analyze the observed fluorescent data using SigmaPlot (Systat Software, Chicago, IL, USA). For fluorescence microscopy, the samples were placed on a microscope slide and covered by a coverslip. Fluorescence images were then taken using a laser scanning microscope (Carl Zeiss, LSM5 Live Duoscan, Germany) equipped with a GFP filter (excitation 488 laser, beam splitter 490, emission LP 505), at a 630× magnification (C-Apochromat).

### 3.4. Measurement of the Formaldehyde Dehydrogenase and Methanol Dehydrogenase Activity Using FA-GESS and Frm-GESS

For the application study of FA-GESS and Frm-GESS, formaldehyde dehydrogenase (FalDH from *Pseudomonas putida*) and methanol dehydrogenase (Mdh from *Bacillus methanolicus*) were used as the model enzymes as in References [30,31,32]. The *falDH* and *mdh* genes were synthesized with codon optimization (Macrogen) and then cloned into the pET28a (+) plasmid (Novagen, Merck, Germany) with a *trc* promoter. The biosensor plasmids pFA-GESS and pFrm-GESS were co-transformed with pET-FalDH and pET-Bsmdh, respectively. To determine the enzymatic activity, pFA-GESS/pET-FalDH and pFrm-GESS/pET-Bsmdh cells were grown in LB medium containing antibiotics (ampicillin 50 μg/mL, kanamycin 25 μg/mL) at 37 °C for 16 h, and then 10% of the grown cells were inoculated in LB medium. After a 2 h incubation, the expression of enzymes was induced by adding IPTG (1.0 mM), and each substrate, specifically 100 μM formaldehyde and 200 mM methanol, was supplied. The fluorescence intensities, corresponding to the production of formate and formaldehyde, were monitored for 10 h after the IPTG induction.

## 4. Conclusions

In this study, we developed a C1-detectable whole-cell biosensor that could detect formaldehyde, formate, and methanol, which could allow the precise determination of C1-converting enzymes inside living cells. These C1-detectable biosensors can also be used to detect the activity of C1-converting enzymes by measuring the fluorescence intensity. To expand this further, the biosensors incorporating the C1-converting enzymes could detect the starting substrate through an enzymatic reaction. These whole-cell biosensors represent a powerful screening system for novel C1 gas-derived chemical-converting enzymes or organisms from both the metagenomic libraries and different natural environments.

## Figures and Tables

**Table 1:** *E. coli* strains and plasmids used in this study.

Name	Description	Reference
***Strain***		
*E. coli* DH5α	Cloning and protein expression	NEB
***Plasmid***		
pFA-GESS	Amp^R^, P_fhlA_-FhlR-P_fdhF_-sfgfp	This work
pFrm-GESS	Amp^R^, P_frm_-frmR-sfgfp	This work
pMeOH-GESS	Amp^R^, P_frm_-frmR-sfgfp, P_trc_-Bsmdh	This work
pET-trc	Kan^R^, trc promoter	This work
pET-Bmmdh	Kan^R^, P_trc_-Bmmdh	This work
pET-Faldh	Kan^R^, P_trc_-Faldh	This work
pFA-Frm-GESS	Amp^R^, FA-GESS, Frm-GESS module	This work
pAC-Bsmdh-Faldh	Cm^R^, P_BAD_-Bsmdh, P_rham_-Faldh	This work

**Table 2:** Characteristics of the C1 biosensors in response to formate, formaldehyde, and methanol addition.

Name	Minimal Detection Concentration (mM)	Operational Range (mM)	Fold-Change
FA-GESS	1.0	1.0–250	7
Frm-GESS	1.0 × 10^−3^	1.0–50 × 10^−3^	12
MeOH-GESS	25	5–400	5

## Supplementary Materials

The following are available online at https://www.mdpi.com/1422-0067/20/9/2253/s1. Table S1. Oligonucleotides used in this study.

## Abbreviations

Mdh	Methanol dehydrogenase
FalDH	Formaldehyde dehydrogenase
Bmmdh	Methanol dehydrogenase from *B. methanolicus*
Bsmdh	Methanol dehydrogenase from *B. stearothermophilus*
FalDH	Formaldehyde dehydrogenase from *P. putida*
MMOs	Methane monooxygenase
IPTG	Isopropyl β-d-1-thiogalactopyranoside
TF	Transcription factor
