# Supplementary material for: C1 Compound Biosensors: Design, Functional Study, and Applications
Source: Int J Mol Sci. 2019 May 7;20(9):2253. doi: 10.3390/ijms20092253 (PMC6540204; doi:10.3390/ijms20092253)
Supplement: Supplementary file 1 [file ijms-20-02253-s001.pdf]

Table S1. Oligonucleotides used in this study

Name	Sequence (5'-3')	Reference
FhlR F	TGCGTCATCGCCATTGGTACCTTAAATCAATGCCGATTTATC	pFA-GESS
FhlR R	CCCAGCGATTTCATCAACAAGCTTGATTTACCACACGCCGAA	
PfdhF F	TTCGGCGTGTGGTAAATCAAGCTTGTTGATGAAATCGCTGGG	
PfdhF R	GTTCTTCACCTTTGCTCATCGGTCTCGCTCCAGTTAATCAA	
FA-v-F	TTGATTAACTGGAGCGAGACCGATGAGCAAAGGTGAAGAAC	
FA-v-R	GATAAATCGGCATTGATTTAAGGTACCAATGGCGATGACGCA	
PfrmR F	GAGTGCACCAGTTATATACTATAGGGGGGTATGC	pFrm-GESS
PfrmR R	CCTTTGCTCATCTCTCGCTCTTCCTCAATATGGT	
Frm-v-F	GAGCGAGAGATGAGCAAAGGTGAAGAACTGT	
Frm-v-R	TATAGTATATAACTGGTGCACTCTCAGTACAATCTG	
Frm-v1-F	ATTGTCAAGCAGATTGTACTGAGAGTGCAC	pMeOH-GESS
Frm-v1-R	AGAAGATTAATCTGATGCCGCATAGTTAAGC	
trc-Bsmdh F	CGGCATCAGATTAATCTTCTTTCAGTTTCAGAACGATACG	
trc-Bsmdh R	AGTACAATCTGCTTGACAATTAATCATCCGGCTCG	
pET-fal v F	CGCAGCATAATGACTCGAGGATCCGGCTG	pET-Faldh
pET-fal v R	TTCCCGACATGGTTTAACCTCCTGTGTGAAATTGTTATC	
Faldh F	AGGTTAAACCATGTCGGGAAACCGCGG	
Faldh R	CCTCGAGTCATTATGCTGCGGAAAAGGTCTTGTG	
mcherry-Frm F	AGAGCGAGAGATGGTGAGCAAGGGCGAG	pFrm-GESS-
mcherry-Frm R	AAACAGAAGCCTACTTGTACAGCTCGTCCATG	mcherry
Frm-v2-F	GTACAAGTAGGCTTCTGTTTTGGCGGATG	
Frm-v2-R	TGCTCACCATCTCTCGCTCTTCCTCAATATGGTAATAG	
FA-gess m F	AGACGGTCACTTCTTCGTCTGTTTCTACTGGTATTGG	pFA-Frm-GESS
FA-gess m R	CAGACAAGCTAAGAGTTTGTAGAAACGCAAAAAGG	
Frm-gess m F	ACAAACTCTTAGCTTGTCTGTAAGCGGATG	
Frm-gess m R	AGACGAAGAAGTGACCGTCTCCGGGAG	pAC-Bsmdh-
Pbad-Bsmdh F	AACAGGAGGAATTAACCATGAAAGCGGCGGTTGT	Faldh
Pbad -Bsmdh R	CTGCAGGTCGACTCTAGAGGTACCTTAATCTTCTTTCAGTT	
Prham F	GGTACCTCTAGAGTCGACCTGCAGCAAGATACAGCGTGAAT	
Prham R	CCGCGGTTTCCCGACATATGGTGATCCTGCTGAA	
Prham Faldh F	TTCAGCAGGATCACCATATGTCGGGAAACCGCGG	
Prham Faldh R	CAGCCAAGCTTGCATGCTTATGCTGCGGAAAAGG	
pAC-v-F	CCTTTTCCGCAGCATAAGCATGCAAGCTTGGCTG	

pAC-v-R	ACAACCGCCGCTTTCATGGTTAATTCCTCCTGTT	
